# Power Reshapes the Mental Representation of Social Hierarchy

**DOI:** 10.3390/bs16081428

**Published:** 2026-08-19

**Authors:** Yan Xi, Fengxiao Hao, Xiaorong Cheng, Xianfeng Ding, Zhao Fan

**Affiliations:** 1 School of Psychology, Central China Normal University, Wuhan 430079, China; xiy@mails.ccnu.edu.cn (Y.X.); hfx522@mails.ccnu.edu.cn (F.H.); x.cheng@ccnu.edu.cn (X.C.); 2 Key Laboratory of Adolescent Cyberpsychology and Behavior (CCNU), Ministry of Education, Wuhan 430079, China; 3 Key Laboratory of Human Development and Mental Health of Hubei Province, Wuhan 430079, China

**Keywords:** power, social hierarchy, psychological disparity, eye movement

## Abstract

Social hierarchy represents a ubiquitous principle of social organization, often intuitively conceptualized as a pyramidal structure. The present study investigated whether subjective power can reshape this mental representation, thereby attenuating perceived psychological distance between social classes. Using eye-tracking technology and time-series representational similarity analysis, we measured participants’ gaze patterns toward stimuli denoting high- and low-status individuals under different power states. Results revealed a robust upward gaze bias toward high-status targets in the control condition, consistent with a pyramid-like representation in which upper classes occupy the apex and lower classes the base. This bias was amplified in the powerless condition but significantly attenuated in the powerful condition, suggesting that subjective power transforms the internalized hierarchy into a more egalitarian configuration, wherein social classes are perceived as more equivalent. Furthermore, power states systematically modulated horizontal gaze position and pupil diameter. These findings provide novel insights into how subjective power dynamically reshapes the representation of social hierarchy and point to new theoretical avenues for reducing perceived inequality at the psychological level.

## 1. Introduction

*Social Hierarchy* was first scientifically described by Norwegian scientist Thorleif Schjelderup-Ebbe in 1921, who proposed that such a hierarchical structure reduced intense conflicts and injuries, saved energy, and promoted social stability (F. Wang et al., 2014). It represents a ubiquitous principle of social organization across a broad range of species, from ants and fish to birds, primates, and humans. The capacity to accurately infer one’s own social position, as well as that of others, within a social hierarchy is critical for successful social interaction and psychological and physical health (Chiao, 2010; F. Wang et al., 2014). In general, social hierarchies are relatively rigid, and upward mobility across social strata is notoriously difficult to achieve over a short timeframe (Beller, 2009; Hertel & Groh-Samberg, 2014; Xie et al., 2022).

*Power* is also a fundamental concept in the social sciences and typically defined as the ability to exert influence or control over others by providing resources (Emerson, 1962; Fiske, 1993; Galinsky et al., 2003; Keltner et al., 2003). It could be mapped onto our mind to form subjective power (the sense of power), which refers to individuals’ perception of their ability to influence others (Anderson et al., 2012). Power is inherently intertwined with social hierarchy. Specifically, higher social class is typically associated with greater power, whereas lower social class is linked to diminished power.

Notably, discussions of power and social class frequently employ spatial metaphors to convey hierarchical relationships. For instance, we call the royalty “*Your highness*”. The most powerful person is usually at the *top* of a hierarchical pyramid of society, and the powerless underlings are at the *bottom*. People will *look up to* those bigwigs of the hierarchical pyramid. A large body of empirical evidence has demonstrated that individuals mentally represent the concepts of power and social hierarchy using vertical spatial dimensions. Specifically, high power or social class is mentally mapped onto upper vertical space, whereas low power or social class is associated with lower vertical space (Chiao, 2010; Giessner & Schubert, 2007; Lakens et al., 2011; Mason et al., 2014; Zanolie et al., 2012; Zhang et al., 2019). This robust association between power, social hierarchy, and vertical space is posited to stem from both developmental and evolutionary origins (Giessner & Schubert, 2007; Meng et al., 2019; Schubert, 2005).

Given that power and social hierarchy are inherently intertwined, we ask whether enhancing subjective power can restructure the mental representation of social hierarchy and engender psychological equality, even as actual social class proves highly resistant to short-term manipulation. Recent studies have demonstrated that individuals’ sense of power exerts broad-ranging effects on their cognition, emotion, and behavior (Galinsky et al., 2003; Keltner et al., 2003; Krumhuber et al., 2023; Lin et al., 2021; Schwab & Singh, 2024; X. Wang et al., 2018). Specifically, the sense of power shapes individuals’ perceptions or mental representations of the self and others. For instance, when experiencing a high sense of power, individuals exhibit an overestimation of their own physical height (Duguid & Goncalo, 2012) and underestimate others’ physical height (Yap et al., 2013), relative to both those in a low-power state and their actual height. Extending this line of work, Hao et al. (2023) further found that the vertical representation of the self and other is dynamically modulated by one’s power state: the self is mentally elevated to upper space when in a high-power state, but debased to lower space when in a low-power state. Collectively, these findings suggest that if power can alter the vertical representations of the self and others, it is highly likely to reshape individuals’ perceptions and mental representations of social hierarchy. 

To address this question, we employed eye-tracking technology to examine eye movement patterns induced by stimuli representing different social classes across different power conditions: control, powerless, and powerful. A large body of empirical research has established a robust link between spatial mental representations and eye movements. Specifically, eye-tracking constitutes a valuable tool for investigating the dynamics of spatial mental representation, especially in situations where these representations can be altered and reshaped (Fourtassi et al., 2017), even when examining abstract mental representations (Felisatti et al., 2022; Götz et al., 2020; Hartmann, 2015; Viganò et al., 2024). Addressing this question not only offers direct eye-tracking evidence for the hierarchical mental representation of social class, but also sheds further light on the connection between abstract social cognition and oculomotor behavior. Critically, the present study contributes novel empirical evidence for dynamic shifts in the mental representation of social hierarchy across distinct power states.

Therefore, we hypothesize that, in the control condition, given the established association between high/low social class and upper/lower mental space, participants were expected to fixate more on relatively upper/lower space following exposure to high-class/low-class stimuli. In contrast, in the high/low-power condition, we predicted that eye movement patterns would diverge from this baseline. Critically, due to the elevation of the self when experiencing a high sense of power, participants would not exhibit the typical pattern of looking up at high-class others, and the mental pyramid of social hierarchy would collapse.

## 2. Materials and Methods

### 2.1. Participants

Sixty-five college students (28 male) from the local university participated in the experiment with payments. They were 22.3 years old on average (range 19 to 26). All of them were native Mandarin speakers with normal or corrected-to-normal vision. All participants signed a consent form according to the requirements of the Institutional Review Board of the local university.

The sample size was determined based on the previous studies on similar topics (Hao et al., 2023). We also used G*Power to estimate sample size (GPower 3.1.9.2; Faul et al., 2007), at power = 0.95, alpha = 0.05, and assumed medium effect size f = 0.25, which resulted in n = 54 participants (ANOVA, repeated measures, within–between interaction). We recruited 65 in case some participants had to be excluded because they turned out to be outliers. 

### 2.2. Materials and Apparatus

Thirty Chinese words referring to people with different social classes were selected as critical items of the experiment. Ten words referred to people of high class, ten to people of medium class, and ten to people of low class. Stimulus selection involved two stages. First, a panel of social psychology experts generated an initial pool of 60 words (20 per social class level: high, middle, low) based on theoretical frameworks and prior literature. Second, independent raters evaluated each word on a 5-point scale for how strongly it signaled class membership. Then, the expert panel applied inclusion criteria (e.g., high rater agreement, theoretical relevance) and exclusion criteria (e.g., ambiguous or outdated terms) to select the top 10 highest-ranked words for each level, yielding a final set of 30 experimental stimuli. In a pretest, the words were rated by another group of 39 participants on a five-point scale. The difference was significant for three classes (F (2,76) = 579.01, *p* < 0.001, *η_p_*^2^ = 0.938). High-class words (*M* = 4.46, *SD* = 0.27) were rated significantly higher in social hierarchy degree than medium-class words (*M* = 2.65, *SD* = 0.45), *p* < 0.001. Medium-class words were rated significantly higher than low-class words (*M* = 1.16, *SD* = 0.07), *p* < 0.001. 

Two hundred and forty pictures of concrete objects were selected from a standard picture pool (Snodgrass & Vanderwart, 1980). These pictures are black-and-white line drawings created according to a set of rules ensuring consistency in pictorial representation. Each picture was 150 × 150 pixels in size. The 240 pictures were randomly combined into 120 picture pairs. The positions of the pictures in each pair were then switched to produce another 120 pairs, resulting in a total of 240 picture pairs for the experiment. For each pair, one picture was presented in the upper area of the screen with its top-left corner at coordinates (325, 110)^1^ and its bottom-right corner at (475, 260), and the other was presented in the lower area of the screen with its top-left corner at coordinates (325, 340) and its bottom-right corner at (475, 490).

The experiment was implemented using an EyeLink 1000 eye tracker (SR Research, Ottawa, Ont., Canada) and Experiment Builder software (Version 2.3.1). Each word consisted of two Chinese characters and was displayed at the center of a VGA color monitor (37.6 cm × 30 cm) with a 100 Hz refresh rate and 800 × 600 pixel resolution against a white background. The display timing was synchronized with the video refresh cycle. When viewed from a distance of 70 cm, the words subtended angles of approximately 1.35° × 0.76°. The EyeLink1000 system was used to track the right eye at a sampling rate of 1000 Hz. To constrain head movements, a head restraint and a chinrest were used throughout the experiment.

### 2.3. Experimental Design

A 3 × 3 mixed design was employed. The first independent variable, power (powerful, powerless, and control group), served as the between-subjects factor. The second independent variable, social class (high, medium, and low), served as the within-subjects factor. The dependent variables included the screen position of the fixation point after stimulus onset (measured in pixels) and pupil size (measured in pixels).

### 2.4. Procedure and Task

The participants were first required to take the Generalized Sense of Power Scale (GSPS) to test their trait power (Anderson & Galinsky, 2006). The Chinese version of the Generalized Sense of Power Scale (GSPS) used in this study was translated and revised by Yao et al. (2020), and has demonstrated good reliability and validity in Chinese samples (Cronbach’s α = 0.93). Then, they were randomly divided into three groups: the powerful group, the powerless group, and the control group. State power was manipulated using the classic power-priming methods (Anderson & Galinsky, 2006; Galinsky et al., 2003; Hao et al., 2023). Specifically, participants were asked to write down specific events in which they exerted power over others (powerful group) or had power exerted over themselves by others (powerless group). They were instructed to include as many details of the event as possible, including specific feelings experienced. Then, the participants completed the manipulation check to evaluate their state power. The manipulation check required the participants to report whether they felt influential, independent, powerful, unimportant, and subordinate (α = 0.83). This inventory has been used extensively in previous research (Duguid & Goncalo, 2012; Hao et al., 2023; Lammers & Stapel, 2009).

Finally, the participants began the formal eye-tracking experiment. After reading the instructions, the eye-tracking system was calibrated and validated for each participant. Each trial started with the presentation of a central red fixation cross for 500 ms, on which the participant had to fix his or her gaze. The fixation cross was then replaced by a central word (high class, medium class and low class) that lasted for 300 ms. Then, two pictures were presented in the upper and lower areas of the screen simultaneously, and lasted for 2000 ms (Ruiz Fernández et al., 2011). Participants were required to name the word as soon as possible. Then, a blank was presented for 2000 ms until the next trial started. The pictures were independent of the preceding word stimuli. They depicted neutral concrete objects with no social-class or power connotations, and were randomly assigned to trials regardless of the word’s class category. Thus, there was no matching or mixing between word class and picture content.

**Figure 1:**
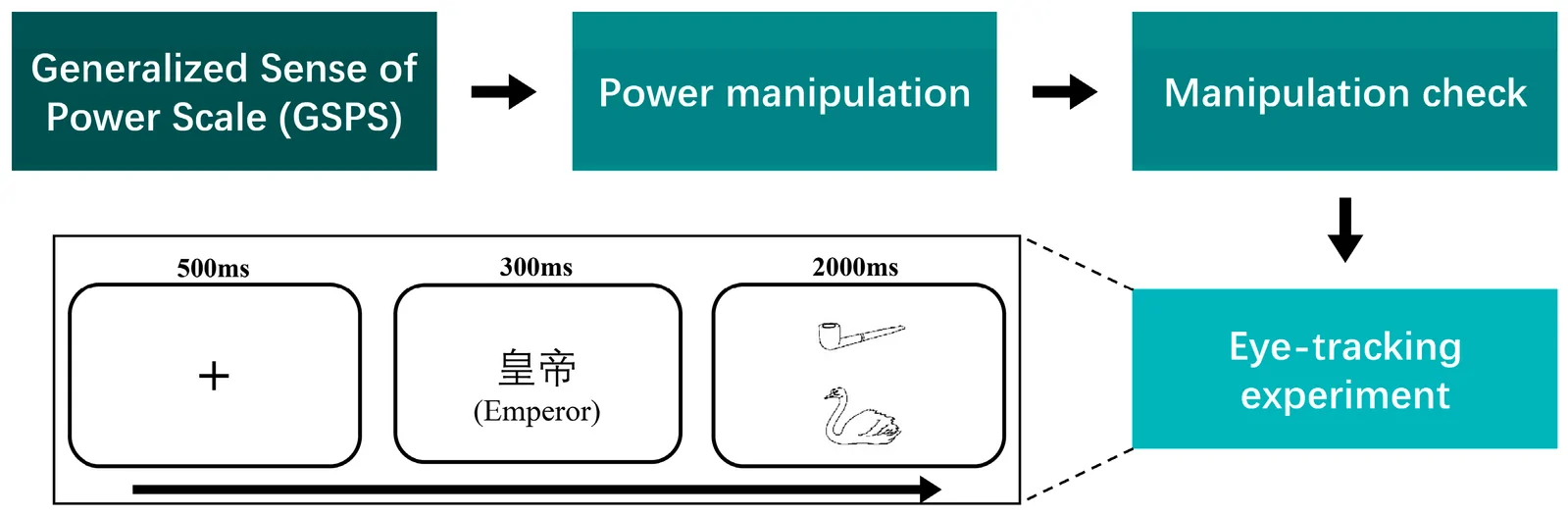
The procedure of experiment.

In a trial, eye movement recording started from the presentation of the word and lasted throughout the subsequent picture display until their removal. The instantaneous x-y coordinates of the right eye were sampled and stored every 1 ms, along with a record of the time-stamped stimulus events on screen. These data were then analyzed offline. The experiment employed three experimental blocks. Each block consisted of 84 trials (4 practice trials and 80 regular trials) separated by short breaks. There was a total of 240 regular trials, with each of the 30 words presented eight times. The total test time lasted for about 25 min (Figure 1).

### 2.5. Data Recording

The eye fixation data after the presentation of the word was collected. In order to exclude invalid saccades or false apparatus feedback, an eye fixation was defined as an eye movement to a screen position with a minimal duration of 100 ms (Bradley et al., 2000; Ruiz Fernández et al., 2011). The screen coordinates were defined as (0, 0) for the top-left corner and (800, 600) for the bottom-right corner. 

### 2.6. Data Analysis

Time-series representational similarity analysis (RSA) was used to examine the dynamic activation of spatial representations of social classes under different power conditions. First, a 50 ms sliding window (step size = 1 ms) was applied to the raw time-series fixation data. Then, the correlation between subjective class evaluation scores and fixation positions at each time point following class stimulus onset was analyzed (2000 ms). Specifically, a cluster-based permutation test within a linear mixed model (LMM) was conducted using the “Permutes 2.8” package (Voeten, 2023) in the R 4.4.3 environment (R Core Team, 2023). The model EyeData ~ ClassRating * Power + (1+ ClassRating|Participant) + (1+ ClassRating|Item) was employed to assess the main effects of power and class, as well as their interaction. Power (categorical: control, powerless, powerful) and ClassRating (continuous: subjective evaluation score) were treated as fixed effects, whereas Participant and Item were random effects.

Subsequently, if the Power × ClassRating interaction effect was significant, simple effects tests were conducted within each power group using the model EyeData ~ ClassRating + (1 + ClassRating |Participant) + (1 + ClassRating|Item) to identify significant temporal clusters. Finally, to further characterize the class effect within each group, repeated-measure ANOVAs were performed on the peak values of these significant temporal windows, with Class treated as a categorical variable (High, Medium, Low). Bonferroni correction was used to control the family-wise error rate for the pairwise comparisons, and we report Cohen’s d (specifically, d_z, computed as the mean difference divided by the SD of the difference scores) as the effect size.

## 3. Results

### 3.1. Manipulation Check

There were no significant differences in trait power among the three groups of participants (*M_powerful_* =38.77, *SD* = 6.06; *M_powerless_* = 36.50, *SD* = 5.27; *M_control_* = 36.85, *SD* = 5.27) (*F* (2,50) = 1.55, *p* > 0.05). However, the state power was significantly different among the three groups (*F* (2,50) = 16.36, *p* < 0.01, *η_p_*^2^ = 0.482); specifically, the powerful group (*M* = 29.03, *SD* = 2.87) significantly exceeded both the control group (*M* = 17.17, *SD* = 3.12) and the powerless group (*M* = 14.17, *SD* = 5.13), with no significant difference observed between the latter two. These results suggested the success of power manipulation.

### 3.2. Time-Series Representational Similarity Analysis in Y-Axis

**Figure 2:**
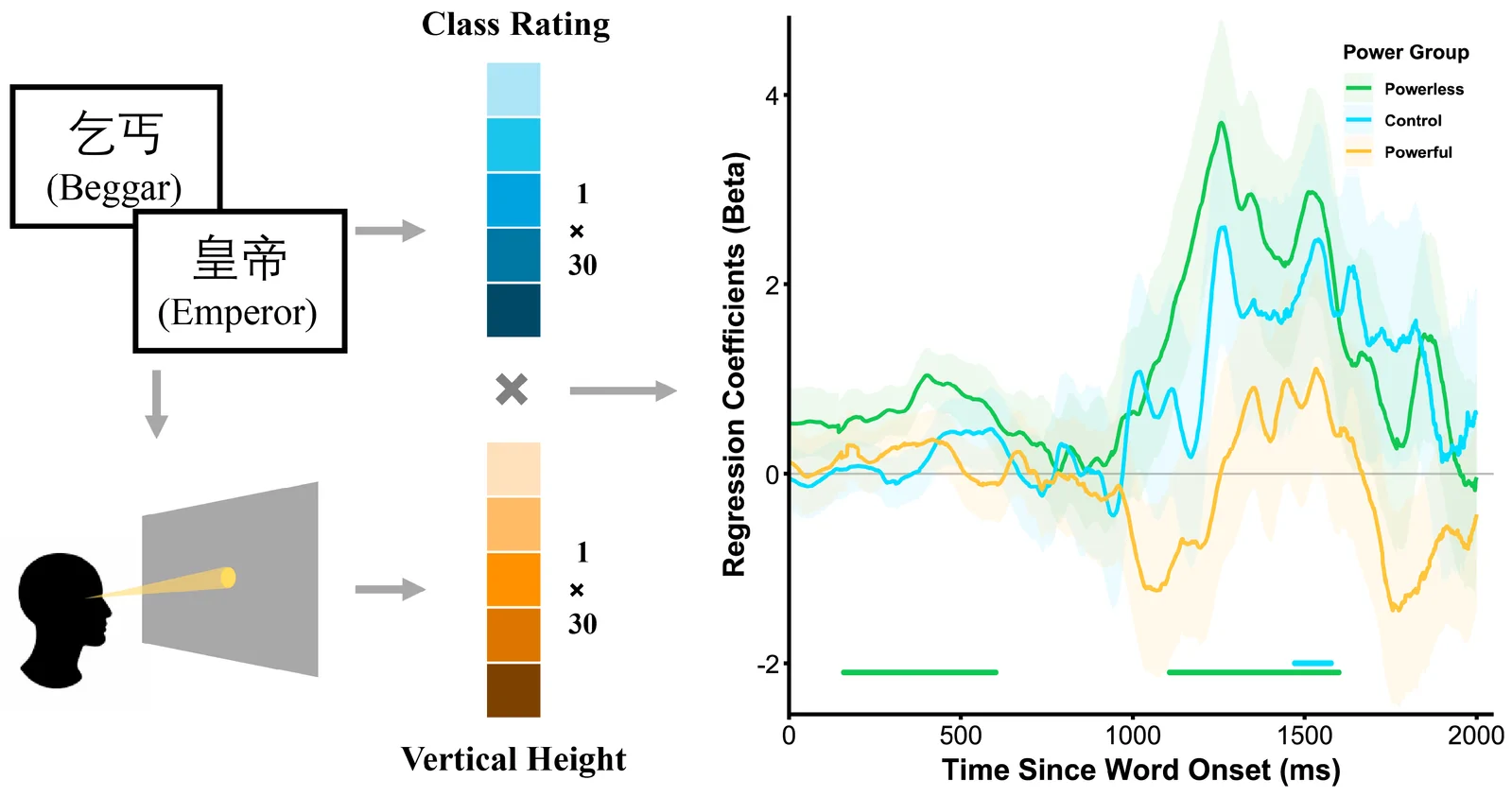
Beta (the regression coefficients between social class ratings and vertical height) as a function of time for three power groups. Lines with different colors represent significant time intervals (*p* < 0.001).

The time-series representational similarity between participants’ social class rating and fixation position (Y axis) in 2 s were analyzed using a Time Series Cluster-based Permutation LMM (Figure 2).

The results showed significant interaction effects between power and class in vertical space. Specifically, significant interactions between power and class were observed between the control and powerless group during the periods of 0–0.5 s; between the powerful and powerless group during the periods of 0.4–0.6 s and 1.0–1.9 s; and between the control and powerful group during the periods of 1.0–1.1 s and 1.7–1.9 s (*ps* < 0.001). As these interaction effects were significant, separate simple effect analyses were conducted for classes within each power condition.

**Figure 3:**
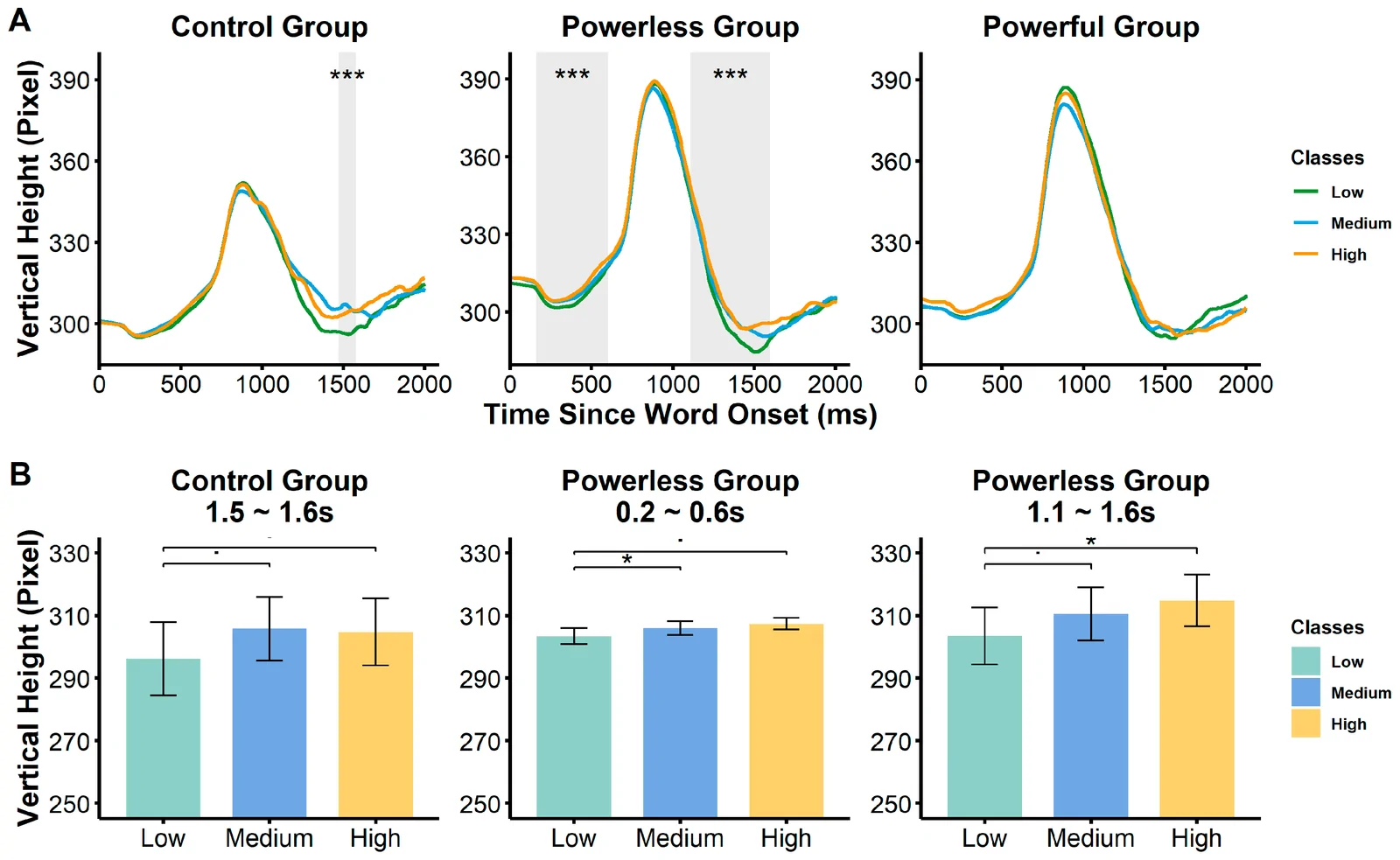
(**A**) Fixation positions as a function of class and time, separately for three power groups. The shaded areas represent significant time intervals (***: *p* < 0.001). (**B**) Peak points of different classes and power groups within significant time intervals. Error bars represent standard errors of means (*: *p* < 0.05).

For the *control* condition, the results indicated a significant positive correlation between social class rating and vertical height during the period of 1.47–1.58 s (*β* = [1.83, 2.47], *se* = [1.22, 1.24], *p* < 0.001) (Figure 2). The higher the social class score, the higher we look up in vertical space. Furthermore, a repeated-measure ANOVA of peak point of the significant temporal cluster revealed a significant effect between three social classes (*F* (2,30) = 4.681, *p* = 0.017, *η_p_*^2^ = 0.238). Pairwise comparisons with Bonferroni correction revealed that fixation positions were higher for the high-class than for the low-class condition (*t*(15) = 2.35, *p* = 0.098, *Cohen’s d* = 0.588), and also higher for the medium-class than for the low-class condition (*t*(15) = 2.58, *p* = 0.062, *Cohen’s d* = 0.646) (Figure 3). These results suggested that individuals tend to elevate their gaze when presented with words associated with high class, demonstrating a relative “look up”.

For the *powerless* condition, the results indicated significant positive correlations between class rating and vertical height not only during the late period of 1.1–1.6 s as in the control condition (*β* = [1.54, 3.71], *se* = [1.03, 1.11], *p* < 0.001) but also during the early period of 0.16–0.6 s (*β* = [0.49, 1.04], *se* = [0.26, 0.40], *p* < 0.001) (Figure 2).

Repeated-measure ANOVA within the *early* significant temporal cluster revealed a significant effect between three social classes (*F* (2,44) = 5.405, *p* = 0.008, *η_p_*^2^ = 0.197). Further comparison indicated that the fixation positions of the high class were higher than that of the low class (*t*(22) = 2.50, *p* = 0.061, *Cohen’s d* = 0.521); the fixation positions of the medium class was significantly higher than that of the low class (*t*(22) = 2.62, *p* = 0.047, *Cohen’s d* = 0.546) (Figure 3). 

Analysis within the *later* significant temporal cluster also revealed a significant effect between three social classes (*F* (2,44) = 5.869, *p* = 0.006, *η_p_*^2^ = 0.211). Further comparison indicated that the fixation positions of the high class were significantly higher than that of the low class (*t*(22) = 3.307, *p* = 0.018, *Cohen’s d* = 0.633); the fixation positions of the medium class were higher than that of the low class (*t*(22) = 2.49, *p* = 0.062, *Cohen’s d* = 0.519) (Figure 3). 

For the *powerful* condition, however, the results indicated no significant correlation between social class score and vertical height during the entire 2 s period.

### 3.3. Time-Series Representational Similarity Analysis in X-Axis

**Figure 4:**
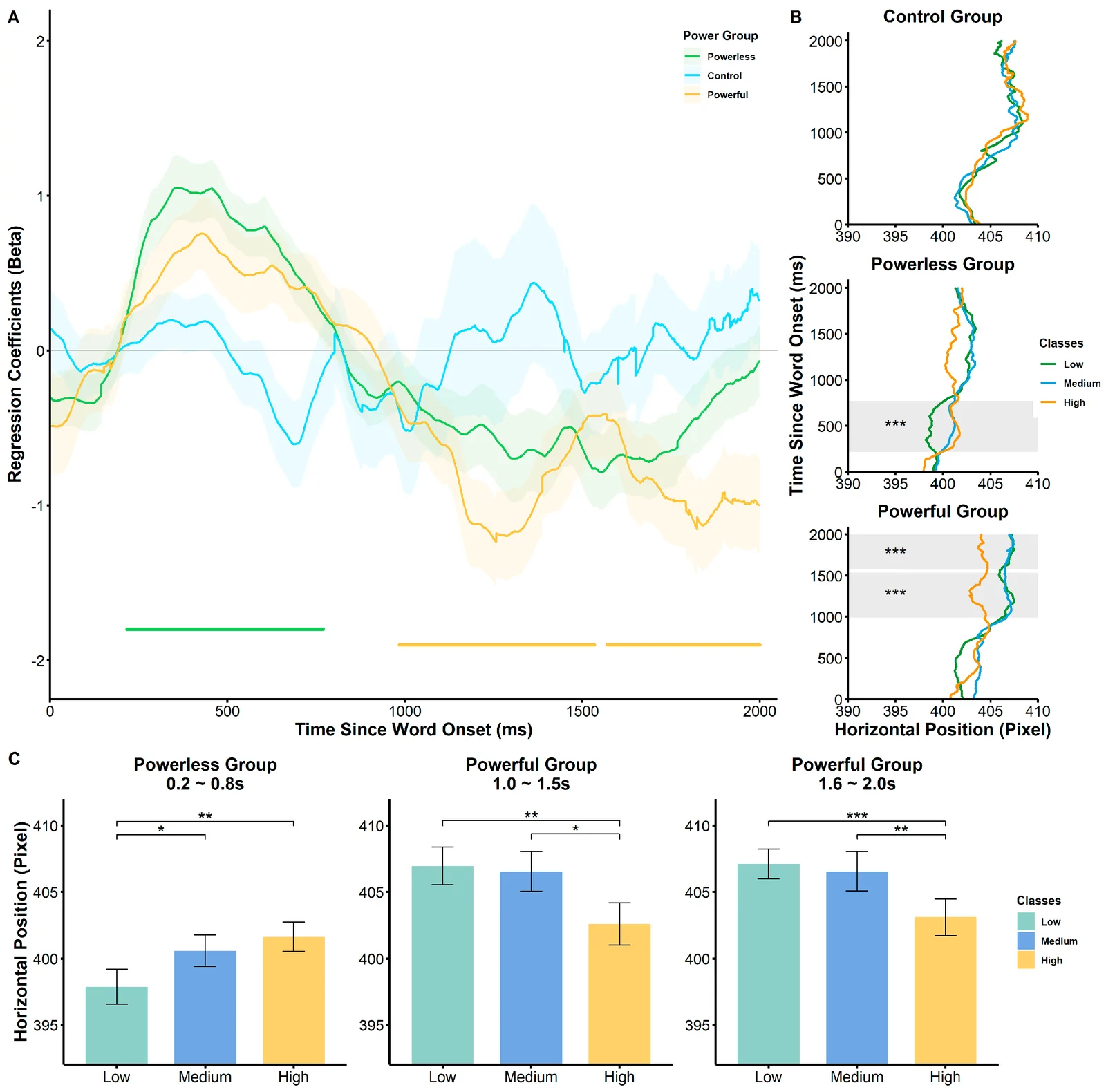
(**A**) Beta (the regression coefficient between social class score and horizontal position) as a function of time for three power groups. The lines with different colors represent significant time intervals. (**B**) Horizontal position as a function of class and time, separately for the three power groups. The shaded areas represent significant time intervals. (**C**) Peak points of different classes and power groups within significant time intervals (*: *p* < 0.05; **: *p* < 0.01; ***: *p* < 0.001).

The time-series representational similarity between participants’ social class scores and fixation positions on the X axis were analyzed in the same way as that on the Y axis. The results showed significant interaction effects between power and class in horizontal space. Specifically, significant interactions between power and class were observed between the control and powerful groups during the periods of 0.3–0.78 s and 1.1–2 s; between the control and powerless groups during the periods of 0.22–0.77 s and 1.12–1.8 s; and between the powerful and powerless groups during the periods of 0.25–0.4 s and 1.17–1.29 s and 1.77–2 s (*ps* < 0.001). As these interaction effects were significant, separate simple effect analyses were conducted for classes within each power condition (Figure 4).

For the *control* condition, however, the results indicated no significant correlation between social class score and horizontal position during the entire 2 s period. For the *powerless* condition, the results indicated a significant *positive* correlation between social class score and horizontal position during the period of 0.22–0.77 s (*β* = [0.23, 1.05], *se* = [0.15, 0.22], *p* < 0.001) (Figure 4A,B). The higher the social class score, the more right the participants look. Repeated-measure ANOVA within the significant temporal cluster revealed a significant effect between three social classes (*F* (2,44) = 11.222, *p* < 0.001, *η_p_*^2^ = 0.338). Further comparison indicated that the horizontal position of the high class was significantly more right than that of the low class (*t*(22) = 4.033, *p* = 0.002, *Cohen’s d* = 0.841); the horizontal position of the medium class was significantly more right than that of the low class (*t*(22) = 2.827, *p* = 0.029, *Cohen’s d* = 0.590) (Figure 4C).

For the *powerful* condition, however, the results revealed a significant *negative* correlation not only during the period of 0.98–1.53 s (*β*= [−1.24, −0.37], *se* = [0.25, 0.29], *p* < 0.001), but also during the period of 1.57–2 s (*β*= [−1.15, −0.45], *se* = [0.28, 0.31], *p* < 0.001) (Figure 4A,B). The higher the social class score, the more left the participants look.

Repeated-measure ANOVA within the period of 0.98–1.53 s revealed a significant effect between three social classes (*F* (2,50) = 9.729, *p* < 0.001, *η_p_*^2^ = 0.280). Further comparison indicated that the horizontal position of the high class was more left than that of the low class (*t*(25) = −3.449, *p* = 0.006, *Cohen’s d* = −0.676); the horizontal position of the medium class was also was more left than that of the low class (*t*(25) = −3.131, *p* = 0.013, *Cohen’s d* = −0.614). Analysis within the period of 1.57–2 s also revealed a significant effect between three social classes (*F* (2,50) = 13.279, *p* < 0.001, *η_p_*^2^ = 0.347). Further comparison indicated that the horizontal position of the high class was more left than that of the low class (*t*(25) = −5.296, *p* < 0.001, *Cohen’s d* = −1.039); the horizontal position of the medium class was also more left than that of the low class (*t*(25) = −3.486, *p* = 0.005, *Cohen’s d* = −0.684) (Figure 4C).

### 3.4. Time Series Analysis of Pupil Size

**Figure 5:**
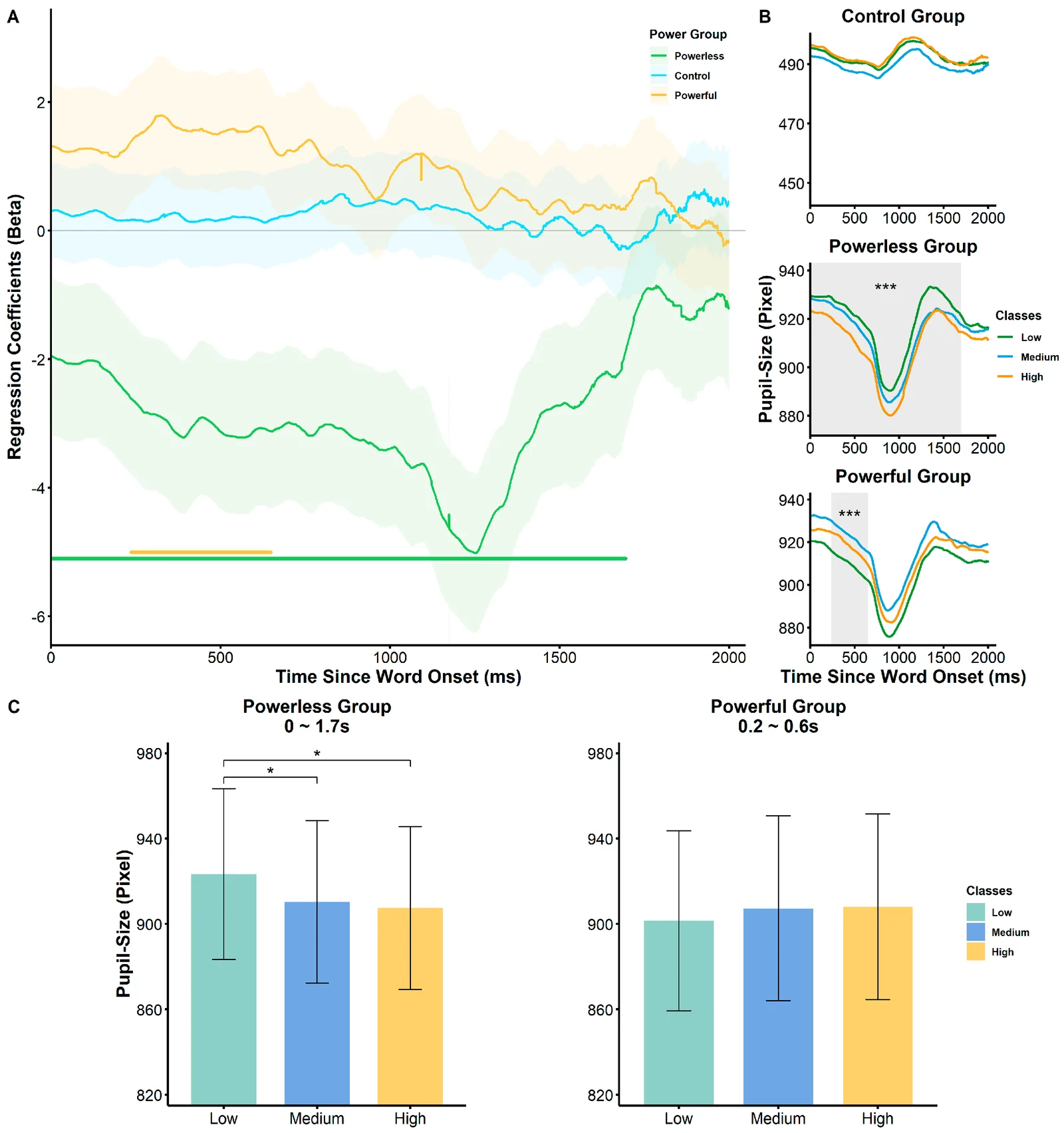
(**A**) Beta (the regression coefficient between social class score and pupil size) as a function of time for three power groups. Lines with different colors represent significant time intervals. (**B**) Pupil size as a function of class and time, separately for the three power groups. The shaded areas represent significant time intervals. (**C**) Peak points of different classes and power groups within significant time intervals. (*: *p* < 0.05; ***: *p* < 0.001).

Participants’ pupil size in 2 s was analyzed using a Time Series *Cluster-based Permutation* LMM. The results showed significant interaction effects between power and class. Specifically, significant interactions between power and class were observed between the control and powerless groups during the periods of 0–1.7 s, and between the powerful and powerless groups during the period of 0–1.8 s (*ps* < 0.001). As these interaction effects were significant, separate simple effects analyses were performed with the model (Figure 5).

For the *control* condition, the results indicated that participants did not exhibit any significant difference in pupil size across different classes. For the *powerless* condition, the results indicated a significant *negative* correlation between social class score and pupil size during the period of 0–1.7 s (*β* = [−5.01, −1.87], *se* = [1.19, 2.15], *p* < 0.001) (Figure 5A,B). The higher the social class score, the smaller the pupil size. 

Repeated-measure ANOVA within the significant temporal cluster revealed a significant effect between three social classes (*F* (2,44) = 8.036, *p* = 0.001, *η_p_*^2^ = 0.268). Further comparison indicated that the pupil size of the high class was significantly smaller than that of the low class (*t*(22) = −3.159, *p* = 0.014, *Cohen’s d* = −0.659); the pupil size of the medium class was significantly smaller than that of the low class (*t*(22) = −2.813, *p* = 0.03, *Cohen’s d* = −0.587) (Figure 5C).

For the *powerful* condition, however, the results revealed a significant *positive* correlation during the period of 0.24–0.65 s (β = [1.35, 1.79], *se* = [0.91, 0.94], *p* < 0.001) (Figure 5A,B). The higher the social class score, the bigger the pupil size. Repeated-measure ANOVA within the temporal cluster revealed a marginally significant effect between three social classes (*F* (2,50) = 2.805, *p* = 0.07, *η_p_*^2^ = 0.101) (Figure 5C).

## 4. Discussion

The present study investigated whether enhancing subjective power could reshape the mental pyramid of social hierarchy. Specifically, we examined how power modulates eye movement patterns subsequent to exposure to words that convey information about different social classes. This approach enables us to explore dynamic changes in the mental representation of social classes across varying power states.

The results revealed a significant positive correlation between social class score and vertical height in the control condition. Individuals’ fixation positions for high- and middle-class stimuli were significantly higher than those for low-class stimuli. This pattern reflected a typical class effect, wherein high social class was associated with relatively upper space and low social class with relatively lower space. In the powerless state, this class effect was similar to that in the control condition: fixation positions for high- and middle-class stimuli were significantly higher than those for low-class stimuli in both the early (0.16–0.6 s) and late (1.1–1.6 s) time windows. However, the typical class effect was completely abolished in the powerful state.

These findings not only provide direct eye-tracking evidence for the hierarchical mental representation of social class, but further elucidate the intimate link between abstract concept and eye movement. Eye-movement patterns in the control and powerless conditions revealed a pyramid-shaped representation of social class. This pattern aligns with the common experience of metaphorically “looking up to” high-status individuals and “looking down on” low-status others. These results converge with prior work establishing eye-tracking as a valuable tool for investigating the dynamics of spatial mental representations, particularly in situations where these representations can be altered and reshaped (Felisatti et al., 2022; Fourtassi et al., 2017; Götz et al., 2020; Hartmann, 2015; Viganò et al., 2024). Furthermore, while a large body of research has documented that *concrete* concept comprehension is grounded in sensorimotor activation (Gallese & Lakoff, 2005; Fischer & Zwaan, 2008), empirical evidence linking *abstract* concepts to sensorimotor processes remains relatively limited. The present research extends this research by demonstrating a direct association between abstract social hierarchy representations and involuntary eye movements, thereby providing novel insights into the embodied cognition of abstract concepts.

More importantly, the present study provides novel empirical evidence for dynamic shifts in the mental representation of social hierarchy across distinct power states. In the powerless state, the pyramidal representation was similar to that in the control condition, and even more pronounced. This pyramidal class effect emerged in both the early stage (0.16–0.6 s after the first saccade) and the late stage (1.1–1.6 s), suggesting that the pyramidal representation was rapidly activated under the powerless condition. However, the eye-movement pattern in the powerful condition diverged sharply from that in the control condition. The typical class effect (high class–upper space/low class–bottom space) vanished: the internal pyramidal representation of social class was reconstructed into an egalitarian structure in which all social classes were mentally represented as equivalent. This finding aligns with prior research examining the relationship between power and the representation of the self and others (Duguid & Goncalo, 2012; Hao et al., 2023; Yap et al., 2013). When individuals were in a powerful state, the self was mentally elevated to a high-status position, and their perceptions and representations of others were correspondingly altered—such that they no longer exhibited the typical tendency to “look up to” high-status individuals.

Notably, an alternative interpretation of the above findings under the powerful condition deserves consideration. According to Fiske’s (1993) attentional account, power might reduce overall attention to hierarchical cues rather than reflect a genuine shift toward more egalitarian social perception. However, if that were the case, we would expect the powerful condition to exhibit distinct global patterns in gaze behavior compared with both the powerless and control conditions. Contrary to this prediction, the overall eye-movement trajectories—along both the vertical and horizontal axes—were remarkably similar across powerful and powerless states. Pupil-size patterns under the two conditions also showed close correspondence. This convergence implies that the effect does not arise simply from inattention to class-related cues; rather, it is more consistent with the view that powerful states modify the spatial representation of social class. Thus, we propose that power influences social perception not by diminishing attentional engagement, but by reshaping the spatial representation through which social class is encoded.

Interestingly, we also observed that the association between social class and horizontal gaze position was modulated by power state. Specifically, in the control condition, social class did not significantly predict gaze position. However, in the powerless state, higher-class stimuli were associated with a more rightward gaze bias, whereas in the powerful state this pattern reversed, with higher-class stimuli predicting a more leftward gaze bias. A possible explanation, grounded in an embodied cognition framework, is that the vast majority of people, who are right-handed, implicitly associate “good” with the right space and “bad” with the left space (Casasanto, 2009). Given that high social status is typically linked to goodness or strength, and low status to badness or weakness, high status becomes naturally associated with the right side of space, and low status with the left. Furthermore, paralleling the vertical spatial mapping of hierarchy, a powerless state may intensify the activation of this horizontal spatial mapping, while a powerful state may alter the observer’s spatial self-representation, thereby reversing the spatial mapping of hierarchy. These findings warrant further investigation in future research.

The present study also revealed power-related modulations of pupil dynamics. Specifically, there was a significant interaction between power manipulation and social class. In the control condition, there were no significant differences in pupil size between social class groups. In contrast, in the powerless condition, participants in the high social class exhibited significantly smaller pupil sizes compared to those in the low social class from the early stage of stimulus presentation. In the powerful condition, this pattern was attenuated or even reversed. A plausible explanation for these findings is rooted in embodied cognition theory: Pupillary constriction occurs when participants mentally simulate bright environments or process words associated with brightness (Laeng & Sulutvedt, 2014; Mathôt et al., 2017). Thus, in the powerless state, the self is figuratively diminished to a lower hierarchical position, prompting individuals to mentally “look up” to high-status others positioned at the top of the social class pyramid. This upward-oriented mental simulation may induce perceived brightness, thereby resulting in pupil constriction.

Several limitations of the present study should be acknowledged. *First*, our sample was relatively small and demographically narrow, cautioning against broad generalization; future replication studies should recruit larger, more diverse populations. *Second*, the analyses of horizontal eye movements and pupil size were exploratory rather than confirmatory in nature. As such, the observed effects of power on these measures require further interpretation and should be more systematically integrated with the results obtained from the vertical axis. *Third*, the controlled laboratory paradigm, while ensuring internal validity, limits ecological generalizability. Future research could adopt naturalistic approaches such as experience-sampling methods, mobile eye-tracking in real-world social encounters, or computational analyses of social media language, which would provide more ecologically valid evidence of class-related attentional processes. *Finally*, while our paradigm does not directly address covert power, future studies could usefully examine whether more implicit power manipulations—such as those embedded in social roles or non-verbal contexts—produce similar or differential effects on social-hierarchy gaze coupling. Moreover, alternative perspectives, such as Foucault’s analysis of power as a diffuse, embodied, and agonistic force (Foucault, 1977), offer a complementary lens that emphasizes the micro-politics of social relations and the potential for resistance.

In conclusion, individuals typically conceptualize social class as a hierarchical pyramid, which is further accentuated in a state of powerlessness. In contrast, enhancing subjective power attenuates the tendency to “look up to” high-status others, and the mental social class pyramid collapses. These findings suggest that social class representations are malleable rather than fixed, providing a useful starting point for future research to examine whether and how such perceptual shifts may translate into broader social outcomes.

## Data Availability

The full dataset used for analysis and all necessary code are available at https://doi.org/10.5281/zenodo.17735585.

## Note

1 Pixels served as both the unit of eye movement analysis and the unit for screen coordinates. These coordinates were defined as the distance in pixels from the top-left corner of the screen (x-axis, y-axis).
